# Paraseptal Lucencies Mimicking Emphysema in a Non-smoker With Acute Lung Injury in COVID-19

**DOI:** 10.7759/cureus.71010

**Published:** 2024-10-07

**Authors:** Sreeja Sanampudi, Margaret Kypreos, Sameer Chabbra, Kiran Batra

**Affiliations:** 1 Radiology, University of Texas Southwestern Medical Center, Dallas, USA; 2 Pulmonology and Critical Care, University of Texas Southwestern Medical Center, Dallas, USA; 3 Radiology, Medical College of Wisconsin, Milwaukee, USA; 4 Cardiothoracic Imaging, University of Texas Southwestern Medical Center, Dallas, USA

**Keywords:** covid-19, emphysema, ground-glass opacities, paraseptal emphysema, respiratory failure

## Abstract

Paraseptal emphysema can be smoking-related but has other causes, including surfactant deficiency, COVID-19, and age. The typical acute chest tomographic findings of COVID-19 include bilateral ground-glass opacities with or without consolidation and interstitial thickening in a peripheral and posterior predominant distribution. Evolution of these findings can occur and ultimately lead to fibrosis. The development of bullae, pneumomediastinum, and pneumothorax can occur as complications of non-invasive or mechanical ventilation. This case report describes incidental paraseptal lucencies that mimicked paraseptal emphysema in a patient with acute hypoxemic respiratory failure secondary to COVID-19 without a prior history of smoking only requiring a high-flow nasal cannula.

## Introduction

COVID-19 is an infectious disease caused by the severe acute respiratory syndrome coronavirus 2 and can present with a myriad of symptoms, including shortness of breath, cough, fatigue, and olfactory dysfunction [1,2]. Acute hypoxemic respiratory failure can ultimately ensue and require supplemental oxygen, non-invasive ventilation, or mechanical ventilation. Chest imaging is indicated in patients with moderate-to-severe symptoms, risk of progression due to comorbidities, and worsening of respiratory condition [1]. In typical cases, chest radiography shows bilateral opacities that are predominately peripheral and lower lobe in distribution. The sensitivity of chest radiography is low, and chest computed tomography (CT) may be indicated in conditions where complications are suspected [1]. Typical appearance on CT includes bilateral ground-glass opacities with or without consolidation or visible intralobular lines (crazy paving). Atypical manifestations include lobar consolidation, nodules, cavitation, and pleural effusions which may indicate superimposed bacterial or additional infection [1-3]. Emphysema, pneumomediastinum, pneumothorax, and bullae occur mostly as complications of the disease process. Case reports have described the development of pneumomediastinum and bullae in patients requiring either non-invasive or mechanical ventilation [3-6]. Paraseptal emphysema in patients not requiring any form of positive pressure has infrequently been reported. Additionally, there have been reports of paraseptal emphysema occurring as a result of COVID-19 with a resolution of the emphysematous changes a few months following the COVID-19 infection [7]. Here, we report the case of a patient with acute hypoxemic respiratory failure secondary to COVID-19 only requiring a high-flow nasal cannula who was incidentally found to have paraseptal lucencies mimicking paraseptal emphysema.

## Case presentation

**Figure 1:**
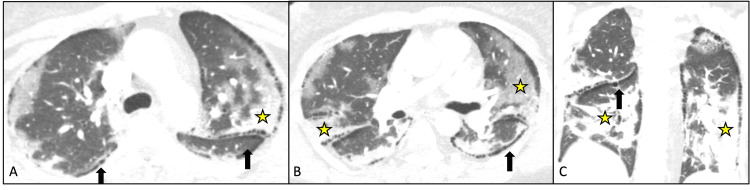
(A, B) CT of the chest axial image at the carina and at the bifurcation at the time of the initial presentation shows bilateral posterior basal predominant consolidative opacities (star). Note paraseptal emphysema-like lucencies along the subpleural space (arrows). (C) CT of the chest coronal image shows peripheral predominant consolidative opacities (star) and paraseptal emphysema (arrow).

A 44-year-old female with no known past medical history of active or passive smoking, connective tissue disease, Marfan syndrome, history of surfactant deficiency, alpha antitrypsin deficiency, or occupational exposure presented with acute hypoxemic respiratory failure secondary to COVID-19. She was diagnosed approximately two weeks before admission and initiated on dexamethasone as an outpatient. Completion of medication as prescribed was questioned, however, was not reinitiated given the number of days from the initial diagnosis. She received one dose of remdesivir and again continuation did not occur due to the number of days from diagnosis. CT of the chest with angiography did not show any evidence of a pulmonary embolism but did show diffuse peripheral ground-glass opacities with peribronchovascular consolidations in the lower lobes. Paraseptal lucencies along the pleura typical of paraseptal emphysema were noted bilaterally throughout both lungs (Figure 1).

**Figure 2:**
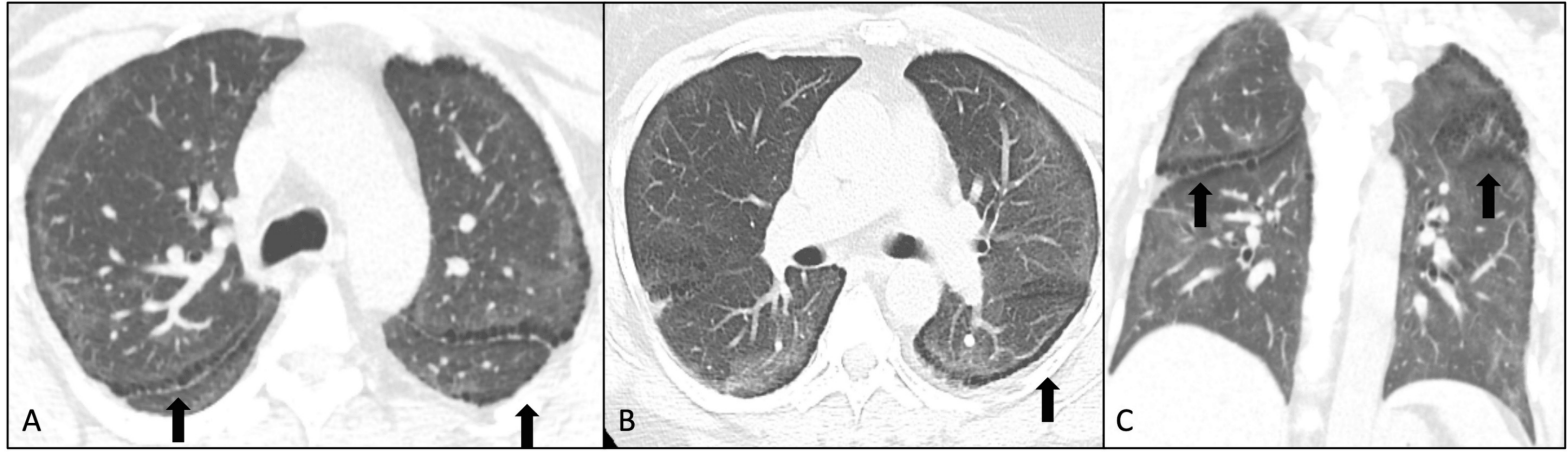
(A, B) High-resolution CT of the chest obtained four months after the initial scan demonstrates the resolution of consolidative opacities with persistent paraseptal emphysema-like lucencies (arrows). (C) CT of the chest shows similar findings along the pleura and the fissures (arrow).

No prior imaging was available for comparison and the patient had never been imaged before this presentation. Initially, she only required a 1 L nasal cannula; however, oxygen requirements quickly escalated and reached a peak of a 9 L high-flow nasal cannula. Pulmonology was consulted and recommended initiation of solumedrol. Doxycycline and ceftriaxone were also initiated for coverage of potential concomitant bacterial pneumonia. Within 24 hours, oxygen requirements improved. Solumedrol taper was initiated, and six days of therapy were completed before transitioning to prednisone. Oxygen requirements continued to improve, and at the completion of the solumedrol taper, she required 4 L of oxygen with exertion. She was discharged on a six-week prednisone taper and oxygen was weaned off by the time of her pulmonary clinic follow-up appointment three weeks later. Repeat high-resolution CT of the chest four months later showed improvement in the ground-glass opacities and consolidations but the persistence of paraseptal lucencies typical of paraseptal emphysema (Figure 2).

## Discussion

Pulmonary emphysema can manifest as centrilobular, paraseptal, and panlobular. Paraseptal emphysema predominately involves the distal alveoli which includes the ducts and the sacs [8]. Typically, paraseptal emphysema is located adjacent to the pleura and septal lines along the periphery of the secondary pulmonary lobule. The majority of the cases of paraseptal emphysema occur from active smoking but other rare causes include passive cigarette smoking, biomass fuels, environmental pollutants such as sulfur dioxide, Marfan syndrome, Ehlers-Danos syndrome, and alpha-1 antitrypsin deficiency [9]. The development of emphysema and bullous lung disease has also been described in patients infected with COVID-19 and secondary to barotrauma after mechanical ventilation [7]. Depending on the clinical course, the diagnosis can be made during the initial hospitalization or on subsequent presentations. Pednekar et al. described a case of extensive bullous lung disease in a patient who was diagnosed with COVID-19 three months earlier. In that particular case, non-invasive positive-pressure ventilation was required during the initial hospitalization and the course was complicated by pneumomediastinum as well as pneumothorax [3]. Alternatively, Xu et al. and Sun et al. described the development of emphysema and bullae throughout their respective patient’s prolonged hospital course [4,5]. In all of these cases, the patients did not have any current or prior tobacco exposure or a family history of alpha-1 antitrypsin deficiency. However, there was a positive history of use of either non-invasive positive-pressure ventilation or mechanical ventilation [3-5]. In our case, the patient did not have any active or passive smoking history, exposure to inhalational drug use, environmental exposure, history of surfactant deficiency, or family history of alpha-1 antitrypsin deficiency. Additionally, non-invasive or mechanical ventilation was not required and paraseptal emphysema was first visualized on the initial CT chest. This case report highlights the incidental finding of typical paraseptal lucencies mimicking emphysema without a prior history of active or passive smoking, connective tissue disorders, or exposure to positive-pressure ventilation in a patient with acute lung injury in the setting of COVID-19.

The pathogenesis of emphysema in the setting of non-smoking or the absence of exposure and presence of infection predominately involves an inflammatory process via three mechanisms [10]. The first mechanism comprises macrophages, neutrophils, and CD8 cells that act on epithelial cells as well as fibroblasts and ultimately lead to parenchymal destruction. The second mechanism involves oxidative stress induced by the inflammatory process. The third mechanism involves an imbalance between pulmonary proteases and antiproteases that are often of genetic origin [10]. However, even if an imbalance is not present, a reduction in the functional activity of the alpha-1 protease inhibitor has been described in patients with acute pneumonia resulting in increased proteolytic damage of lung tissue [3]. It is, thus, possible that, in this case, the significant inflammatory response in the setting of reduced alpha-1 protease inhibitor activity led to the formation of emphysema-like cysts. The persistence of this finding after the resolution of infection remains unclear; however, a case report by Cabanne and Revel reported the resolution of the emphysematous changes with the replacement of a linear band thought to represent organizing pneumonia four months after the initial infection [7]. The pathogenesis of resolution was hypothesized to be secondary to a process known as auto-bullectomy where fibrosis of the walls or obstruction of the airways supplying the bullae occurs [7,11]. In our case, a repeat high-resolution CT of the chest completed four months later showed the persistence of the paraseptal lucencies typical of paraseptal emphysema and resolution of airspace opacities associated with active COVID-19 infection. It is uncertain if there is any causative relationship between COVID-19 infection and the development of paraseptal emphysema and we describe a case of the incidental finding of paraseptal lucencies that mimicked emphysema in a COVID-19 patient with no history of active or passive smoking, connective tissue disease, alpha-1 antitrypsin deficiency, surfactant deficiency or occupational exposure. Further prospective CT chest imaging is needed to assess the association between the above-mentioned findings and COVID-19 with long-term outcomes.

## Conclusions

Paraseptal emphysema-like lucencies may be seen in a non-smoker with no history of passive smoking, surfactant deficiency, connective tissue disorders, or in COVID-19 in the setting of acute hypoxemic respiratory failure not requiring positive-pressure ventilation. Long-term follow-up imaging is required to determine if these findings persist or resolve.

## References

[1] Farias LP, Fonseca EK, Strabelli DG (2020). Imaging findings in COVID-19 pneumonia. Clinics (Sao Paulo).

[2] Kwee TC, Kwee RM (2020). Chest CT in COVID-19: what the radiologist needs to know. Radiographics.

[3] Pednekar P, Amoah K, Homer R, Ryu C, Lutchmansingh DD (2021). Case report: bullous lung disease following COVID-19. Front Med (Lausanne).

[4] Xu W, Luo X, Wang H, Shen C, Song Y, Sun T, Chen M (2021). Pulmonary emphysema, bullae, and pneumothorax in COVID-19 pneumonia. Radiol Case Rep.

[5] Sun R, Liu H, Wang X (2020). Mediastinal emphysema, giant bulla, and pneumothorax developed during the course of COVID-19 pneumonia. Korean J Radiol.

[6] Jagosz M, Guzik W, Moczała Ł, Rydel M, Misiołek H, Białka S (2022). Young convalescent COVID-19 pneumonia with extensive pneumomediastinum emphysema: case report. Clin Case Rep.

[7] Cabanne E, Revel MP (2021). Post-COVID-19 vanishing paraseptal emphysema. Radiology.

[8] Araki T, Nishino M, Zazueta OE (2015). Paraseptal emphysema: prevalence and distribution on CT and association with interstitial lung abnormalities. Eur J Radiol.

[9] Pahal P, Avula A, Sharma S (2023). Emphysema. https://www.ncbi.nlm.nih.gov/books/NBK482217/.

[10] Rakotoson J, Andriamamonjisoa JA, Andriamihary MN, Ratsimbazafy SJ, Randrianarimalala RD, Rakotoarivelo RA, Ralandison S (2021). Giant compressive emphysema: a rare complication of COVID-19. BMC Infect Dis.

[11] Vella-Boucaud J, Chouabe S, Bourin F, Nardi J, Perotin JM, Lebargy F, Deslee G (2014). [Post-infectious autobullectomy]. Rev Mal Respir.

